# Co-transcriptional recruitment of Puf6 by She2 couples translational repression to mRNA localization

**DOI:** 10.1093/nar/gku597

**Published:** 2014-07-09

**Authors:** Karen Shahbabian, Célia Jeronimo, Amélie Forget, François Robert, Pascal Chartrand

**Affiliations:** 1 Département de Biochimie et Médecine Moléculaire, Université de Montréal, 2900 Boulevard Edouard-Montpetit, Montréal, QC, H3C 3J7, Canada; 2 Institut de recherches cliniques de Montréal, 110 Avenue des Pins Ouest, Montréal, QC, Canada; 3 Département de Médecine, Université de Montréal, 2900 Boulevard Edouard-Montpetit, Montréal, QC H3C 3J7, Canada

## Abstract

Messenger RNA (mRNA) localization is coupled to the translational repression of transcripts during their transport. It is still unknown if this coupling depends on physical interactions between translational control and mRNA localization machineries, and how these interactions are established at the molecular level. In yeast, localization of transcripts like *ASH1* to the bud depends on the RNA-binding protein She2. During its transport, *ASH1* mRNA translation is repressed by Puf6. Herein, we report that She2 recruits Puf6 on *ASH1* co-transcriptionally. The recruitment of Puf6 depends on prior co-transcriptional loading of Loc1, an exclusively nuclear protein. These proteins form a ternary complex, in which Loc1 bridges Puf6 to She2, that binds the *ASH1* 3′UTR. Using a genome-wide ChIP-chip approach, we identified over 40 novel targets of Puf6, including several bud-localized mRNAs. Interestingly, the co-transcriptional recruitment of Puf6 on genes coding for these bud-localized mRNAs is also She2- and Loc1-dependent. Our results suggest a coordinated assembly of localization and translational control machineries on localized mRNAs during transcription, and underline the importance of co-transcriptional events in establishing the cytoplasmic fate of mRNAs.

## INTRODUCTION

Localized and temporal control of protein production is one of the mechanisms which has evolved in eukaryotes to regulate events like asymmetric cell division, cell motility and synaptic plasticity (1). To this end, cells employ various and complex mechanisms to control both cytoplasmic localization of specific messenger RNAs (mRNAs) and their local translation (2). While translational repression of mRNAs during their transport has been well established, it is still unclear if these two processes are co-regulated and if the assembly of translational repressors on localized mRNA is physically coupled to the localization machinery. The budding yeast *Saccharomyces cerevisiae* has emerged as a powerful model organism to study this question, since over 20 transcripts are transported from the mother cell and localized to the budding daughter cell (3). One of these transcripts, *ASH1*, is transported to the bud during late anaphase (4). The localization of *ASH1* depends on the co-transcriptional interaction of She2, the main RNA-binding protein involved in bud-localization of mRNAs, with four *cis*-acting elements (or zipcodes) along the mRNA sequence (5). After transcription, the mRNP complex is exported to the cytoplasm, where it is joined by She3 to make it stable and competent for localization (6). She3, on the other hand, bridges the mRNP complex to the molecular motor Myo4, which employs actin filaments to transport this localization complex, or ‘locasome’, to the bud (7).

Beside She2, other RNA-binding proteins like Puf6 and Loc1 have also been identified which are essential for complete localization of *ASH1* mRNA and local synthesis of Ash1. Puf6 was purified from She2-associated mRNPs and is a member of the Pumilio/FBF (fem-3 mRNA binding factor) family of RNA-binding proteins (8). Puf6 directly binds in the 3′untranslated region (UTR) of *ASH1* mRNA and represses the translation of this transcript during its transport (8). Its phosphorylation at the bud tip leads to translational activation and production of Ash1 (9). Loc1 also binds the *ASH1* mRNA 3′UTR and is important for localization and translational control of this transcript (10,11). However, the exact function of this protein is still unknown. Puf6 and Loc1 are mainly nucleolar proteins, suggesting that their interaction with *ASH1* mRNA might take place in the nucleus. Indeed, it was shown that presence of She2 in the nucleus is essential for the association of Puf6 and Loc1 with *ASH1* mRNA and for the translational repression of *ASH1* (12,13). Both Puf6 and Loc1 are thought to be recruited post-transcriptionally during a nucleolar transit of the *ASH1* mRNA mediated by She2 (12). However, both Puf6 and Loc1 are also associated with the transcription elongation factor Spt5 *in vivo* (14), suggesting that Puf6 and/or Loc1 may instead be loaded on mRNA during transcription.

In this study, we used chromatin immunoprecipitation (ChIP) to investigate the recruitment of Puf6 and Loc1 on the *ASH1* mRNA and elucidate the interactions between She2, Puf6 and Loc1. We show that both Puf6 and Loc1 interact with nascent *ASH1* mRNA during transcription and that they require She2 for their co-transcriptional association with this mRNA. We also found that Puf6, Loc1 and She2 form a ternary complex *in vitro* and *in vivo*, and that Loc1 links Puf6 to She2 in this ternary complex. Deletions of Loc1 that disrupt its interaction with She2 result in defective Ash1 sorting to the daughter cell. These findings describe a novel role for Loc1 in physically coupling translational control to the mRNA localization machinery. Finally, using a genome-wide ChIP-chip approach, we detected the co-transcriptional loading of Puf6 on several new mRNA target, including known bud-localized mRNAs, and we show that the recruitment of Puf6 on these transcripts is both She2- and Loc1-dependent. Overall, our results suggest a cooperative recruitment of mRNA localization and translational control factors during the transcription of localized mRNAs.

## MATERIALS AND METHODS

### Yeast strain and DNA manipulation

All yeast strains in this study are derivative of BY4741. Yeast growth was performed in yeast extract peptone dextrose (YPD) or synthetic selective media at 30°C. Polymerase chain reaction (PCR) based gene disruption and protein tagging were performed as described previously (15). Gene disruptions were all confirmed by PCR analysis of genomic DNA. For mutagenesis of Puf6 binding sites (2X UUGU), a two-step PCR-based mutagenesis method was used. Mutated PCR product was cloned into the integrative plasmid YIP128, and the resulted plasmid linearized with EcoRV and transformed into an *ash1* strain. Strains used are listed in Supplementary Table S1.

### Plasmid construction

To construct YCP111-She2MYC and YCP111-She2R63KMYC, She2MYC and She2R63KMYC were PCR amplified from YCP22-She2MYC and YCP22-She2R63KMYC, and cloned into PstI/KpnI sites of YCP111. pGEX-6P1-Loc1 was generated by cloning PCR amplified Loc1 coding sequence into BamHI/NotI sites of pGEX-6P1. pGEX-4T3-Puf6-His was created by amplifying the Puf6 sequence from genomic DNA with primer pairs in which reverse primer contained coding sequence for six histidines. The PCR fragment was then cloned into SalI/NotI sites of pGEX-4T3. YIP128-*ASH1* 3′UTR mutated was constructed by cloning the PCR fragment containing the mutated Puf6 binding sites into the YIP128 BamHI/XmaI sites. To construct pGEM4Z1+mE3, a PCR was performed on genomic DNA from a strain with mutated *ASH1* 3′UTR. The PCR product was then cloned into the HindII/EcoRI sites of the pGEM4Z1 plasmid. Plasmids YCP111-*LOC1*-C1 to C5 are derived from plasmid pRL093, which contains the entire *LOC1* open reading frame with 500 bp of 5′ and 1000 bp of 3′ region, and six myc epitopes inserted at a BamHI site just before the stop codon of *LOC1* (10). First, pRL093 was digested with PstI and SmaI, and the resulting fragment containing the *LOC1* gene was cloned in YCPlac111. The *LOC1* open reading frame with its 5′ sequence was amplified from genomic DNA using primers containing a PstI restriction site on forward primer and three HA tags along with a BamHI restriction site on the reverse primer, to generate the *LOC1*-C1 construct. To generate the *LOC1*-C2 and C3 deletions, the same strategy was used, with the exception that the reverse primers were positioned at +450 and +300 nucleotides from the ATG, respectively. The constructs *LOC1*-C4 and C5 were generated using a two-step PCR strategy, in which the promoter region of *LOC1* was fused to PCR fragments positioned either at +150 or +300 from the ATG, respectively. These PCR products were subsequently cloned in YCPlac111-*LOC1*-C1 digested with Pst1 and BamH1. The sequences of all cloned DNA were verified by Sanger capillary sequencing. Plasmids used in this study are listed in Supplementary Table S2.

### Chromatin immunoprecipitation

For each ChIP, three independent 50 ml early log phase culture was used. All strains were grown in YPD, except for the strain expressing She2WT and She2R63K from YCPlac111, for which selective –leu medium was used. To increase the number of *ASH1* transcripts, cells were synchronized for 2 h using Nocodazole at the final concentration of 15μg/ml. Cells were cross-linked for 5 min at room temperature using formaldehyde at a final concentration of 1%. For quenching of formaldehyde, glycine was added at a final concentration of 0.125 M. Cells were harvested by centrifugation (3000 *g*, 5 min, 4°C) and washed twice with cold phosphate buffered saline (PBS), then resuspended in 1 ml FA lysis buffer (50 mM Hepes/KOH pH 7.5, 140 mM NaCl, 1 mM ethylenediaminetetraacetic acid (EDTA), 1% Triton, 0.1% sodium deoxycholate, 40 U RNasin/ml (Promega), 88 μg/ml phenylmethylsulfonyl fluoride (PMSF), 10 μg/ml leupeptin, 10 μg/ml pepstatin, 5 μg/ml aprotinin) and lysed using glass beads by vortexing five times 30 s with 1 min interval on ice. When RNase treatment was needed, RNase A (four Kunitz units; Sigma) was added to the lysate. Lysed cells were transferred to a new 1.5 ml microtube and sonicated for three times each 20 s, followed by 1 min on ice using 100 Sonic Dismembranator (Fisher Scientific). Debris were removed by centrifugation (16 000 *g*, 5 min, 4°C) and solubilized chromatin was transferred to a new microtube in order to perform immunoprecipitation. Before IP, a 50 μl aliquot of solubilized chromatin was removed and stored at −20°C as ‘input’. Immunoprecipitation was performed overnight at 4°C using 3 μg of each of the following antibodies: total rabbit IgG (Sigma) for TAP-tagged proteins; mouse monoclonal 9E10 antibody (Roche Applied Science) for myc-tagged proteins and 8WG16 monoclonal antibody (Abcam) for immunoprecipitation of RNA Pol II. Immunocomplexes were collected for 4 h at 4°C using 50 μl protein A/G sepharose beads (GE Healthcare). Beads were washed with 700 μl FA lysis buffer, 1 ml of FA 500 buffer (50 mM HEPES/KOH at pH 7.5, 500 mM NaCl, 1 mM EDTA, 1% Triton, 0.1% sodium deoxycholate, 40 U RNasin/ml), 700 μl of LiCl wash (10 mM Tris-Cl at pH 8.0, 250 mM LiCl, 0.5% [v/v] NP-40, 0.1% sodium deoxycholate, 1 mM EDTA), and finally 700 μl of TE/100 mM NaCl. Chromatin was decross-linked and eluted from the beads by heating the beads overnight at 65°C in 25 mM Tris-HCl (pH 7.5), 10 mM EDTA, and 0.5% sodium dodecyl sulphate (SDS). After treatment with 1 mg/ml of Proteinase K (Roche) at 37°C for 2 h, chromatin was phenol extracted and ethanol precipitated and resuspended in 40 μl of TE buffer.

Quantification of the immunoprecipitated and ‘input’ DNA was performed by quantitative real-time PCR by (LightCycler 480 Roche Applied Science) using SsoFast EvaGreen Supermix q-PCR kit (Biorad). Each 20 ul PCR reaction contained 10 μl of quantitative PCR (qPCR) mix, 2μl of DNA and 300 nM of each of the primers. PCR was performed in the following conditions: enzyme activation for 30 s at 95°C, followed by 40 cycles of 5 s at 95°C, 20 s at 57°C for annealing and extension step. Cycle thresholds (Ct) for each triplicate of sample and input were averaged and ChIP enrichment was calculated by dividing the amount of ChIP DNA over input DNA using 2 ^−ΔΔC^_T_ formula. Background represents an amplicon from an intergenic region between the genes YDR539W and YDR540C on Chromosome IV. For ChIP of Rpb1, an amplicon on *SCR1* was used as background. Primers used for ChIP are listed in Supplementary Table S3.

### Chromatin immunoprecipitation-microarrays (ChIP-chip)

ChIP-chip experiments were performed after cross-linking cells with 1% formaldehyde for 30 min as described previously (16), with the following modifications. Puf6 was immunoprecipitated from a strain expressing Puf6-TAP from the *PUF6* endogenous locus. The isogenic non-tagged strain was used as control. Immunoprecipitation was done using 0.5 mg of Dynabeads M-270 Epoxy (Life Technologies) per IP, pre-coupled with rabbit IgG (Sigma) at the concentration of 350 μg of IgG/mg of beads, as per manufacturer's recommendation. In order to preserve interactions mediated via RNA, 40 units/ml of RNAseOUT (Life Technologies) was included in the lysis buffer.

### Peak calling

Significant regions were identified using the algorithm described in (17). The parameters used were as follow: ProbeSetPvalue = 0.0005, Filter1Pvalue = 0.0005, Filter2Pvalue = 0.0005–0.005. This allowed for the identification of 121 regions, overlapping with 117 genes (105 if not counting dubious genes). Each gene was manually curated by two different individuals. After removing dubious and uncharacterized open reading frames (ORFs), targets with ambiguous gene assignments (due to proximal neighboring genes) and genes with no probe enriched at least 2-fold, a list of 43 target genes were retained for further analyses.

### Co-Immunoprecipitation

Yeasts were grown in 100 ml YPD at 30°C to the log phase. Cells were harvested by centrifugation (3000 *g*, 5 min, 4°C) and resuspended in 800 μl of lysis buffer (20 mM Hepes pH 7.5, 20% glycerol, 150 mM NaCl, 0.2 mM EDTA, 0.05% NP-40, 0.1 M beta-Mercaptoethanol). Cells were lysed by vortexing in presence of glass beads for five times 30 s with an interval of 1 min on ice. After centrifugation (16 000 *g*, 5 min, 4°C), the supernatant was transferred to new microtube for performing immunoprecipitation. For Puf6-TAP immunoprecipitation, 5 μg of total IgG (Sigma) was added to lysates and incubated 4 h at 4°C, then 50 μl of Protein G sepharose beads (GE healthcare) was used for the collection of immunocomplexes. Beads were washed three times with wash buffer (20 mM Hepes pH 7.5, 20% glycerol, 200 mM NaCl, 0.2 mM EDTA, 0.05% NP-40), and immunocomplexes were eluted from the beads by heating the beads for 10 min at 95°C in 100 μl of 25 mM Tris-HCl, pH 7.5, 10 mM EDTA, 0.5% SDS.

### Protein purification

Recombinant She2-GST and Loc1-GST over-expression was performed in *Escherichia coli* BL21 cells transformed with pGEX-6P1-She2 and pGEX-6P1-Loc1 plasmids, respectively. For overproduction of Puf6-GST-His, pGEX-4T3-Puf6-His plasmid was transformed into *E. coli* Rosetta cells. Cells were grown in Luria–Bertani (LB) to the mid-log phase and protein induction was performed overnight at 15°C using 1 mM final concentration of Isopropyl beta-D-1-thiogalactopyranoside (IPTG). Cells were harvested by centrifugation (15 000 *g*, 15 min, 4°C) and resuspended in Tris 50 mM pH 8, 5 mM EDTA, 1 M NaCl, complete protease inhibitor cocktail (Roche), 1 mg/ml lysozyme and RNase A at 15 ug/ml final concentration. Lysate was incubated on ice for 30 min, and then sonicated. Soluble proteins were separated from debris by centrifugation (16 000 *g*, 15 min, 4°C) and supernatant was used for affinity chromatography with glutathione sepharose beads (GE healthcare). Beads were washed four times with wash buffer (Tris 50 mM pH 8, 5 mM EDTA, 1.5 M NaCl). Proteins were eluted from beads using reduced glutathione (Sigma) at a final concentration of 10 mM in PBS. Whenever needed, GST tag was cleaved by either PreScission protease (GE healthcare) (for She2-GST and Loc1-GST) or Thrombin (GE healthcare) for Puf6-GST, following instructions provided by manufacturer. For Pug6-GST-His, a second round of purification with His-tag was performed to eliminate unspecific contaminants from glutathione beads purification. After purification by Ni-NTA agarose beads (Qiagen), proteins were eluted from beads using imidazole at a final concentration of 250 mM.

### *In vitro* transcription

Using T7 RNA polymerase (Promega), E3, E1 and mE3 RNAs were transcribed *in vitro* from linearized plasmids pRL168, pGEM4Z1-E1 and pGEM4Z1-mE3, respectively. RNA was phenol extracted and ethanol precipitated and stored in diethylpyrocarbonate (DEPC) H_2_O at −80°C.

### *In vitro* pull-down experiments

For the interaction between Loc1-GST:She2 and Puf6-GST:She2:Loc1, first GST-tagged proteins Loc1-GST and Puf6-GST were bound to glutathione beads, then equimolar quantities of each of the proteins were added to the binding reaction in binding buffer (50 mM HEPES-KOH, pH 7.5, 150 mM NaCl, 2 mM MgCl_2_, 2 mM EDTA, 1 mM DTT, and 5% glycerol). Reaction was incubated for 4 h at 4°C then washed three times with binding buffer. The protein complex was eluted from the beads by heating at 95°C in Laemmli buffer and then separated on 10% Sodium dodecyl sulphate-polyacrylamide gel electrophoresis (SDS-PAGE).

For *in vitro* reconstitution experiments, physiological concentration of each of the proteins was used (see text). First, *in vitro* transcribed RNA was denatured by heating at 65°C for 10 min following by a rapid cooling on ice. RNA was renatured for 15 min at room temperature then incubated with She2 at 4°C for 30 min, in binding buffer in presence of Heparin at final concentration of 1μg/μl. The She2:RNA complex was added to Puf6-His bound to the Ni-NTA agarose beads. When Loc1 was part of the complex, Puf6-His bound to the beads was incubated with Loc1 in binding buffer for 4 h at 4°C to form Puf6:Loc1 complex prior to the addition of She2:RNA complex. For the interaction of Puf6-His with RNA, Puf6-His bound to the beads was added directly to renatured RNA. Beads were washed three times in binding buffer and the reactions were aliquoted in two tubes, one part was used for analyzing eluted proteins by heating beads in Laemmli buffer at 95°C, and the other aliquot was used for purification of pulled-down RNA by heating beads at 65°C in 100 μl of 50 mM Tris-HCl, pH 8.0, 100 mM NaCl, 10 mM EDTA and 1% SDS. For RNA gel, a denaturing 8% PAGE was used, followed by GelRED staining.

### Northern blotting

To extract RNA from yeast, 50 ml of yeast cells in YPD were grown to log phase and harvested by centrifugation (3000 *g*, 5 min, 4°C). Yeast pellet was resuspended in 1 ml cold water and transferred to a 1.5 ml microtube. After centrifugation (3000 *g*, 5 min, 4°C), the pellet was resuspended in 400 μl Tris-EDTA-SDS (TES) solution (10 mM Tris-Cl pH 7.5, 10 mM EDTA, 0.5% SDS) then 400 μl acid phenol was added and incubated at 65°C for 1 h. After centrifugation (16 000 *g*, 10 min, 4°C), 400 μl chloroform was added to the supernatant. After centrifugation (16 000 *g*, 10 min, 4°C), RNA was ethanol precipitated from supernatant and resuspended in 50 ul H_2_O. For northern blotting, 15 μg of total RNA was separated on a 1.5% denaturing agarose gel containing formaldehyde for 4 h in MOPS-EDTA buffer. RNAs were transferred on Nylon membrane (GE healthcare) using capillary blotting. RNAs were UV cross-linked and hybridized with specific *ASH1* probe at 65°C in Church hybridization buffer (1% bovine serum albumin, 1 mM EDTA, 0.5 M NaHPO_4_ pH 7.2, 7% SDS). *ASH1* probe was internally labeled by [α-^32^P] dCTP using Ready-To-Go DNA Labeling Beads (Amersham). *SCR1* probe was an end labeled oligonucleotide, complementary to the middle of *SCR1* sequence, labeled by [γ-^32^P] ATP using T4 polynucleotide kinase.

## RESULTS

### Puf6 and Loc1 are recruited on *ASH1* mRNA during transcription

**Figure 1.**
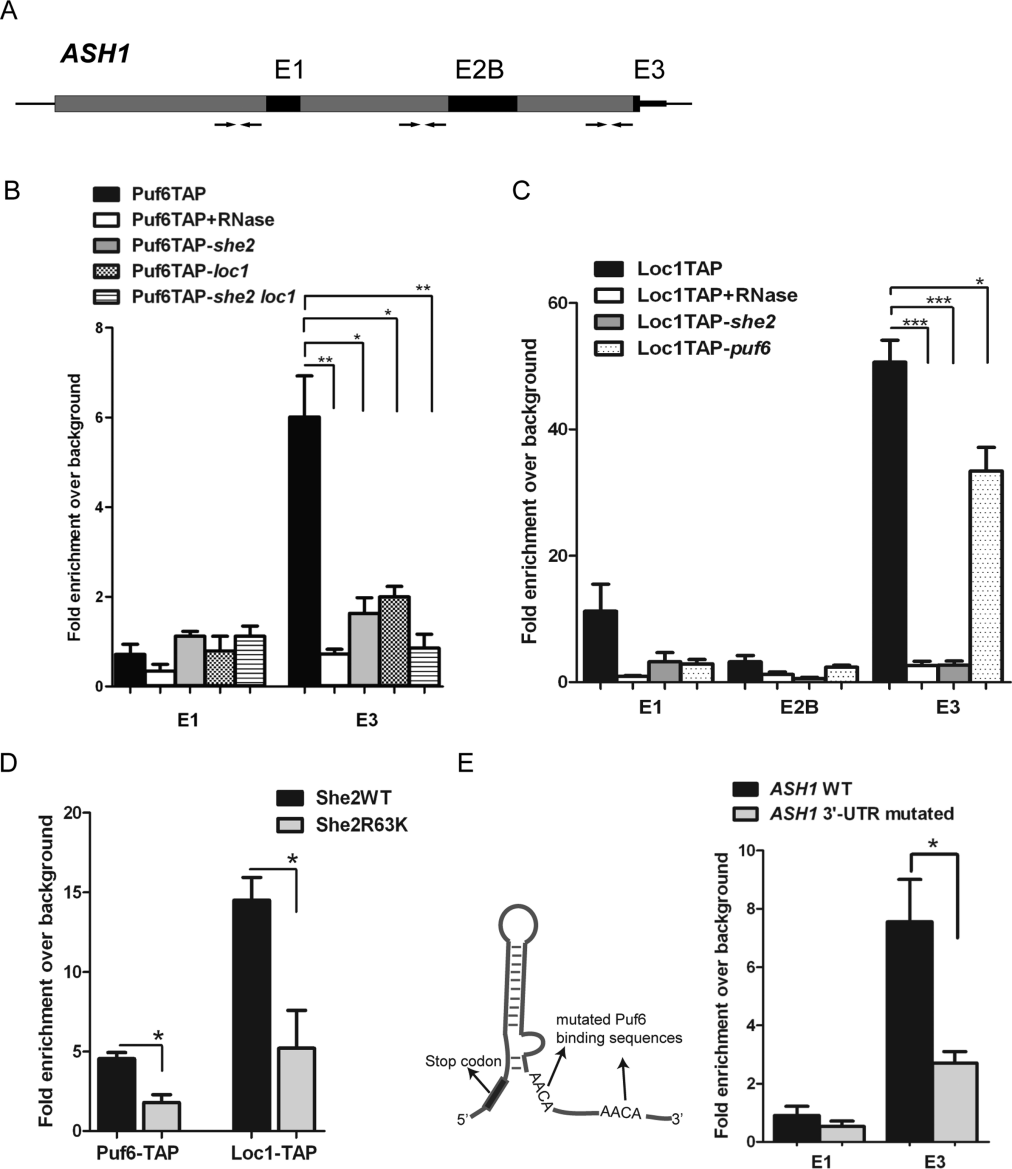
Puf6 and Loc1 are recruited co-transcriptionally on *ASH1* mRNA. (**A**) Schematic presentation of the *ASH1* gene. Dark boxes represent localization elements. Arrows represent amplicons used for qPCR analysis. Amplicon lengths for E1, E2 and E3 are 101 bp, 128 bp and 101 bp, respectively. (**B**) ChIP of Puf6-TAP in WT and mutant strains. After immunoprecipitation of Puf6-TAP, amplicons corresponding to E1 and E3 were amplified. For RNase treatment, chromatin was treated with RNase A prior immunoprecipitation. (**C**) ChIP of Loc1-TAP in WT and mutant strains. After ChIP, three amplicons E1, E2B and E3 were analyzed by qPCR. (**D**) ChIP of Puf6-TAP and Loc1-TAP in strains expressing WT or an RNA-binding mutant of She2 from a centromeric plasmid. After ChIP recruitment of Puf6-TAP and Loc1-TAP on E3 localization element were analyzed by qPCR. (**E**) ChIP of Puf6-TAP in a strain with mutated Puf6 binding sequence in the 3′UTR of *ASH1* mRNA. At left is a schematic presentation of the E3 localization element with two UUGU sequences mutated to AACA. After performing ChIP in WT and mutated *ASH1*, E1 and E3 amplicons were analyzed by qPCR. Values presented are mean ± SEM (*N* = 3) (* *P* < 0.05, **P < 0.01, ***P < 0.001, unpaired *t*-test).

We have recently shown that the She2 localization factor is loaded co-transcriptionally on nascent *ASH1* mRNA (5). Previous studies have also shown that the presence of She2 in the nucleus is essential for recruitment of nucleolar proteins Puf6 and Loc1 on *ASH1* mRNA (12,13). These results raised the hypothesis that She2 may recruit Puf6 and Loc1 during transcription of *ASH1*. To test this possibility, ChIP was performed using a strain expressing an endogenous C-terminal TAP-tagged Puf6. In this assay, chromatin associated with Puf6 was immunopurified, and the enrichment of specific regions of the *ASH1* gene was measured by qPCR. Two specific amplicons from the *ASH1* gene were analyzed: one corresponding to the localization element E3, which contains the Puf6-binding domain, and one corresponding to the E1 localization element, as an internal negative control (Figure 1A). As shown in Figure 1B, the E3 amplicon was specifically enriched after Puf6 ChIP, while there was no enrichment for the E1 amplicon, suggesting an association of Puf6 with the E3 element on the *ASH1* gene. To determine whether this association is direct or mediated via the nascent *ASH1* RNA, chromatin was treated with RNase prior to Puf6 immunoprecipitation. As expected, enrichment of chromatin near element E3 was abolished after RNase treatment, suggesting a co-transcriptional recruitment of Puf6 on nascent *ASH1* mRNA and validating that our ChIP protocol efficiently detects RNA-mediated binding events (Figure 1B).

The same approach was used to determine whether Loc1 is also recruited co-transcriptionally on the *ASH1* mRNA. Results from three-hybrid assays suggest that Loc1 interacts with localization elements E1 and E3 of the *ASH1* mRNA (10). Therefore, after ChIP in a Loc1-TAP strain, the enrichment of three amplicons on the *ASH1* gene was quantified: two amplicons near the localization elements E1 and E3, and one near the element E2B, which serves as an internal negative control (Figure 1A). Chromatin from both elements E1 and E3 was specifically enriched after Loc1-TAP ChIP, but not from the element E2B (Figure 1C). However, a stronger signal was observed at localization element E3, suggesting that Loc1 interacts primarily with this region of the *ASH1* gene. After repeating ChIP in the presence of RNase, Loc1 ChIP signal disappeared for all the regions of *ASH1* (Figure 1C), suggesting an RNA-dependent enrichment of Loc1 on the *ASH1* gene.

Since She2 is important for Loc1 and Puf6 binding to the *ASH1* mRNA in the nucleus (13), the role of She2 in the co-transcriptional recruitment of Puf6 and Loc1 on the *ASH1* gene was tested by ChIP in a *she2* background. For both Puf6 and Loc1, a strong decrease in the enrichment of amplicon E3 after ChIP was observed in *she2* strains compared to wild type (WT) (Figure 1B and C). Decrease of the element E1 amplicon was also observed after Loc1 ChIP in the *she2* background. These results suggest that She2 is necessary for the recruitment of both Puf6 and Loc1 on the nascent *ASH1* mRNA. Since Puf6 and Loc1 interact *in vivo* (11), the interdependency between Puf6 and Loc1 in their recruitment on *ASH1* was also tested. Surprisingly, a 3-fold decrease of Puf6 ChIP signal on the *ASH1* E3 element was observed in a *loc1* strain (Figure 1B), suggesting that Puf6 recruitment is dependent on Loc1. Moreover, when Puf6 ChIP was performed in a *she2 loc1* strain, no enrichment of *ASH1* E3 region was observed (Figure 1B), showing that Puf6 requires both She2 and Loc1 for its recruitment on the nascent *ASH1* mRNA. Concerning Loc1, while its enrichment on E3 element in a *puf6* strain decreased significantly, still nearly 70% of WT enrichment was observed. A reduction was also observed for the association of Loc1 with the E1 element in a *puf6* background (Figure 1C).

To eliminate the possibility that the observed decrease in ChIP signals of Puf6-TAP and Loc1-TAP in the different mutants was caused by an alteration in *ASH1* mRNA abundance, RNA polymerase II occupancy on the *ASH1* gene was measured in the various mutants by performing ChIP of Rpb1, the largest subunit of the RNA polymerase II. No significant differences were observed in Rpb1 ChIP enrichment along the *ASH1* gene in *she2*, *loc1* or *puf6* strains, compared to WT (Supplementary Figure S1A), suggesting that *ASH1* transcription is not affected in these mutants. Northern blot on RNAs extracted from *she2*, *loc1*, *puf6* and *she2 loc1* strains also confirmed that *ASH1* mRNA levels did not vary between these mutants (Supplementary Figure S1B). Moreover, western blot analysis confirmed that Puf6-TAP and Loc1-TAP expression was similar in mutants and WT strains (Supplementary Figure S1C). Altogether, these results show that Puf6 and Loc1 are loaded on the 3′UTR of the nascent *ASH1* mRNA during its transcription. Interestingly, the loading of Loc1 depends on She2, while the loading of Puf6 depends on both She2 and Loc1, suggesting a stepwise assembly of these factors on the nascent transcript.

### She2 RNA binding and PUF binding element are important for the recruitment of Puf6 on nascent *ASH1* mRNA

The results presented above suggest that She2 promotes the recruitment of Loc1 and Puf6 on nascent *ASH1* mRNA. To further confirm the primary role of She2 in this recruitment, ChIP of She2-myc in WT, *loc1* and *puf6* strains was performed. No significant differences were observed for the enrichment of She2 on the localization element E3 of *ASH1* gene in mutant versus WT strains (Supplementary Figure S2), suggesting that She2 interacts with the nascent *ASH1* E3 localization element independently of Loc1 and Puf6. She2 is an RNA-binding protein which directly interacts with stem-loop structures in *ASH1* localization elements (18). Moreover, She2 is associated with the transcription elongation factor Spt4-Spt5 before its recruitment on the localization elements of *ASH1* (5). To investigate which of these capacities of She2 is important for the recruitment of Loc1 and Puf6, a mutant of She2 (She2R63K) that lacks the ability to bind RNA but preserves its interaction with Spt4-Spt5 was tested (5). Puf6-TAP *she2* and Loc1-TAP *she2* strains were transformed with centromeric plasmids expressing either WT She2-myc or mutant She2R63K-myc, ensuring endogenous expression level of She2. ChIP of Puf6-TAP and Loc1-TAP was performed, and enrichment of the E3 amplicon was determined by qPCR. For both Puf6 and Loc1 ChIPs, more than 2-fold decrease in the enrichment level of E3 was observed when the mutant form of She2 was expressed (Figure 1D), suggesting that the recruitment of Puf6 and Loc1 on the nascent E3 localization element relies on the RNA-binding capacity of She2.

Puf6, like other members of the Puf family of proteins, is an RNA-binding protein and recognizes two UUGU tetranucleotides sequences near the localization element E3 in the 3′UTR of *ASH1* (8). These sequences are important for *in vivo* association of Puf6 with *ASH1* mRNA, and asymmetrical distribution of Ash1 (8). To determine whether the Puf6-binding site is necessary for the co-transcriptional recruitment of Puf6, the two UUGU elements located in the 3′UTR of *ASH1* mRNA were mutated to AACA (Figure 1E). Either the WT or mutated *ASH1* gene was integrated at the *LEU2* locus of a Puf6-TAP *ash1* yeast strain. Puf6-TAP ChIP was performed on mutated and WT *ASH1* 3′UTR strains, which revealed a more than 2-fold decrease in the enrichment of Puf6-TAP on the mutated E3 localization element compared to WT 3′UTR (Figure 1E). Altogether, these results suggest that Puf6 requires its binding element, and not only She2, in order to be properly loaded on *ASH1* mRNA during transcription.

### Puf6, Loc1 and She2 form a ternary complex

**Figure 2.**
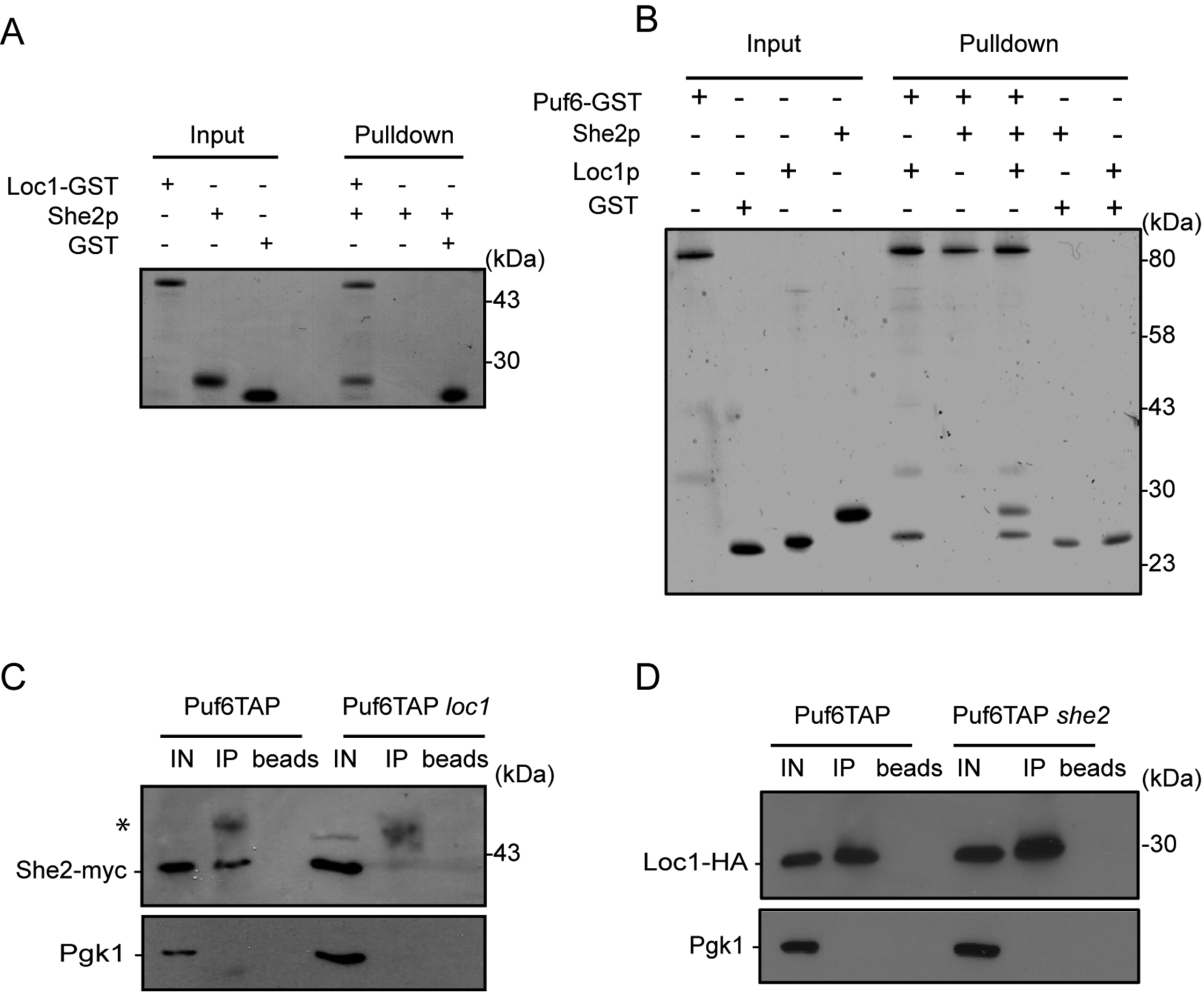
Puf6, Loc1 and She2 interact together and form a ternary complex *in vitro* and *in vivo*. (**A**) GST pull-down experiment to assess direct interaction between Loc1 and She2. Recombinant Loc1-GST and She2 were incubated together and the complex retained on glutathione beads was eluted and analyzed on PAGE by Coomassie blue staining. (**B**) GST pull-down experiment to detect interactions between recombinant Puf6-GST, Loc1 and She2. Puf6-GST is interacting directly with Loc1 but not She2. To detect ternary complex formation, Puf6-GST was incubated with both Loc1 and She2. (**C**) Co-immunoprecipitation assay to detect interaction of Puf6 and She2. Puf6-TAP was immunoprecipitated using total rabbit IgG and the co-purification of She2-myc was verified by performing western blot using rabbit polyclonal antibody to myc epitope. In a *loc1* strain, the interaction between She2-myc and Puf6-TAP is abolished. The asterisk corresponds to IgG heavy chain. (**D**) Co-immunoprecipitation assay to detect interaction of Loc1 and Puf6. Immunoprecipitaion of Puf6-TAP using total rabbit IgG leads to the co-purification of Loc1-HA in both WT and *she2* strains. Western blot was performed using mouse monoclonal HA.11 antibody. All experiments presented were repeated reproducibly three times.

Our results thus far suggest a model in which the interaction of She2 with the *ASH1* mRNA during transcription leads to the recruitment of Loc1 and Puf6 on the E3 localization element. However, direct interaction between these proteins has yet to be reported. To explore possible direct protein interactions among these factors, GST pull-down experiments using recombinant proteins were performed. First, recombinant Loc1-GST and She2 were purified from bacteria and tested for their co-elution from glutathione beads. As shown in Figure 2A, She2 was pulled down from beads containing Loc1-GST but not GST alone, suggesting a direct interaction between She2 and Loc1 *in vitro*. Next, interactions between Puf6-GST and Loc1 or She2 were assessed (Figure 2B). Incubation of Puf6-GST with Loc1 led to the co-elution of the two proteins, indicating a direct Puf6:Loc1 interaction. In contrast, when Puf6-GST and She2 were incubated together, no co-elution was observed, suggesting that there is no direct interaction between Puf6-GST and She2 (Figure 2B). However, when Puf6-GST and She2 pull-down was repeated in presence of Loc1, the complex of three proteins was retained on the beads, showing that Puf6 and She2 form a ternary complex with Loc1 *in vitro*, with Loc1 acting as a bridge between the other two factors.

By revealing this direct interaction between Loc1 and She2, we sought to rule out any possible defect of She2–Loc1 interaction due to the She2R63K mutation in the ChIP experiments presented in the Figure 1D. To this end, recombinant She2(WT)-GST and She2(R63K)-GST were purified from bacteria and their interaction with Loc1 was tested in a GST pull-down assay. As shown in Supplementary Figure S3, the same amount of Loc1 was co-purified from either WT or R63K mutant She2-GST, confirming that this mutation has no effect on the She2–Loc1 interaction.

In order to confirm the role of Loc1 in the formation of a She2-Loc1-Puf6 complex *in vivo*, co-immunoprecipitation experiments using Puf6-TAP and She2-myc were performed. Pull-down of Puf6-TAP led to co-purification of She2-myc (Figure 2C), confirming the interaction between these factors *in vivo* (8,13). However, immunoprecipitation of Puf6-TAP in a *loc1* strain did not result in enrichment of She2-myc in the pellet (Figure 2C), confirming that Loc1 is necessary to bridge Puf6 to She2 *in vivo.* To confirm the interaction between Puf6 and Loc1 *in vivo*, and to determine the role of She2 in this interaction, co-immunoprecipitation was repeated on endogenous Puf6-TAP and Loc1-HA, in either WT or *she2* strains. After immunoprecipitation of Puf6-TAP, Loc1-HA was present in the immunopellet either in presence or absence of She2, suggesting that She2 is not necessary for the interaction between Puf6 and Loc1 (Figure 2D). Altogether, these results show that She2, Loc1 and Puf6 form a ternary complex *in vitro* and *in vivo*, and these data point toward a role for Loc1 in bridging Puf6 to She2.

### *In vitro* reconstitution of E3 localization element mRNP complex

**Figure 3.**
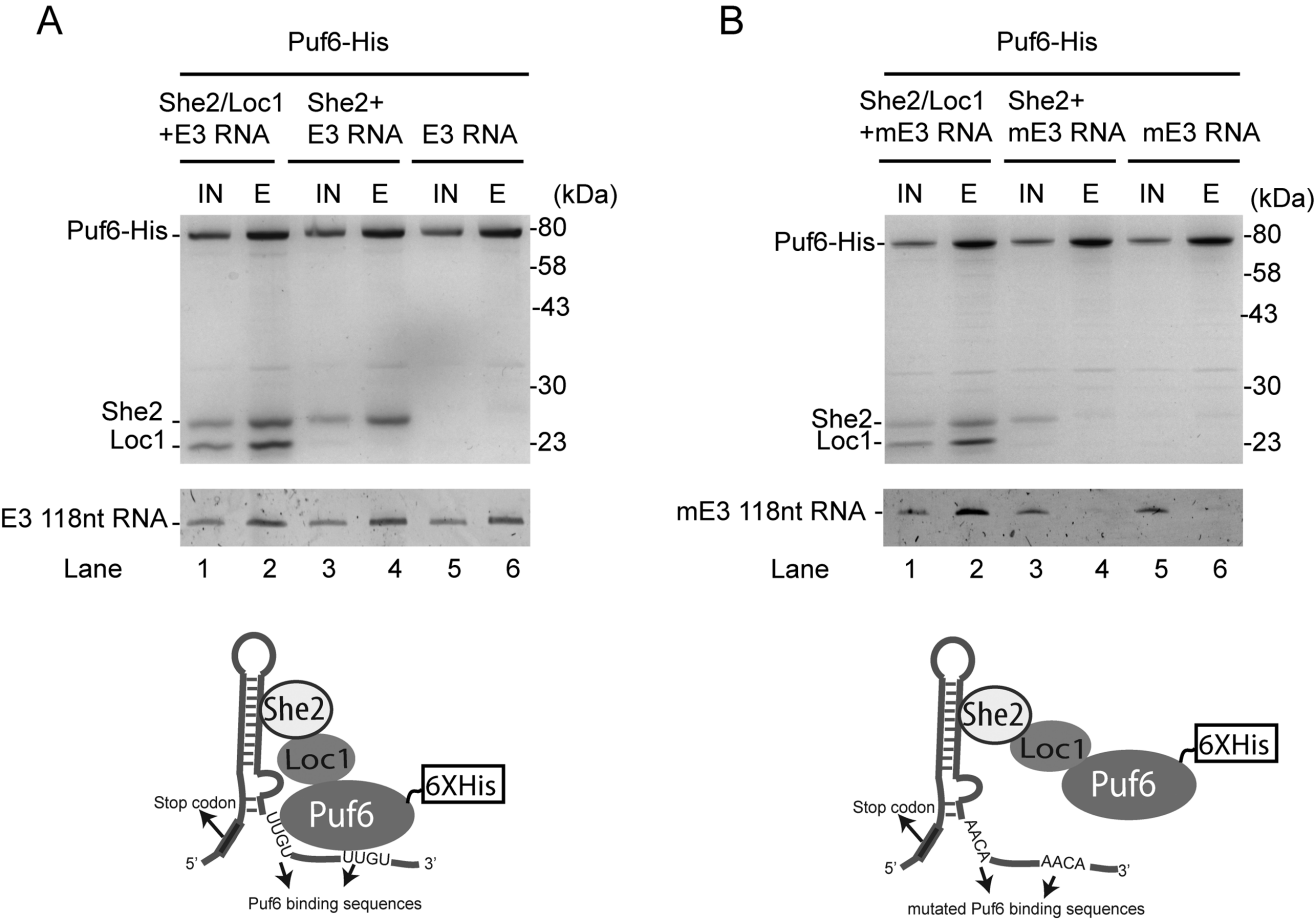
*In vitro* reconstitution assay of E3-mRNP. (**A**) Pull-down experiment with Puf6-His, Loc1, She2 and 118 nt E3 RNA. Upper gel corresponds to protein gel stained by Coomassie blue, lower gel is RNA PAGE stained by Gel-RED (IN = Input, E = Elution). At bottom, cartoon represents schematic interaction between proteins used in the pull-down experiment and E3–118 nt RNA. (**B**) *In vitro* reconstitution assay using E3 RNA with mutated Puf6 binding sequences (mE3). Pull-down experiments performed using Puf6-His, Loc1, She2 and mE3–118 nt. Note that RNA is co-purified with Puf6-His only when ternary protein complex is present. Upper gel is protein gel, lower gel is RNA PAGE. At bottom, shown is schematic presentation of mutated RNA and protein complex.

Although our ChIP results provide evidence for the formation of a multiprotein complex on the *ASH1* E3 element during transcription, these data do not prove that such complexes really assemble. In order to determine whether a Puf6-Loc1-She2 ternary complex can be formed on the *ASH1* E3 RNA *in vitro*, pull-down experiments were performed in presence of a 118 nt E3 localization element RNA using physiological concentration of each protein: 230 nM of She2 (19), 200 nM of Loc1 and 1.2 μM of His-tagged Puf6 (20). The E3 localization element RNA was first incubated with She2, then added to the Puf6-His:Loc1 complex bound to Ni-NTA agarose beads. The Puf6-His:Loc1:She2 complex was co-eluted along with E3 RNA, suggesting that the mRNP complex can be formed *in vitro* (Figure 3A, lane 2). When Loc1 was omitted from the reaction, She2 and the E3 RNA were still co-purified with Puf6-His (Figure 3A, lane 4), suggesting that Puf6 and She2 can also interact via their simultaneous binding to the localization element E3 RNA (6). Finally, in the absence of both She2 and Loc1, Puf6-His was still able to bind the E3 RNA (Figure 3A, lane 6), as previously reported (8).

To further confirm that Puf6 can be recruited on the E3 RNA via its interaction with the Loc1:She2 complex, a mutated version of E3 RNA (mE3), in which the two UUGU sequences that constitute the Puf6-recognition motif were mutated to AACA, was produced *in vitro*. Since the She2-binding element in the mE3 RNA is preserved, this RNA can still bind to She2, but not to Puf6. As expected, the mE3 RNA was not associated with Puf6-His in the pull-down assay in the absence of She2 and Loc1 (Figure 3B, lane 6). However, when pull-down of the Puf6-His:Loc1:She2 ternary complex with this mutated RNA was repeated, the mRNP complex was retained on the beads (Figure 3B, lane 2), suggesting that the RNA-binding affinity of She2 for the mE3 RNA is sufficient to join the Puf6-His:Loc1:She2 protein complex to this RNA. Therefore, one would expect that in the absence of Loc1, the Puf6-His:Loc1:She2 ternary complex would not form, and neither She2 nor the mE3 RNA would bind to Puf6-His. Indeed, when Loc1 was not present in the reaction (Figure 3B, lane 4), both She2 and mE3 RNA were poorly retained on Puf6-coupled beads (compare lane 4 versus lane 2), confirming the role of Loc1 in bridging Puf6 to the She2-mE3 RNA complex.

In order to validate the specificity of the She2-Loc1-Puf6 ternary complex for the E3 element in our *in vitro* assay, its interaction with another *ASH1* localization element (E1) was also tested. Since She2 possesses a lower affinity for the E1 localization element compared to the E3 element (6), and since the E1 RNA does not contain any Puf6-binding motif, this RNA should bind poorly to the ternary complex at the concentration of She2 used in our pull-down experiments. For this experiment, a 70 nt RNA corresponding to the minimal localization element E1 (18) was *in vitro* transcribed and used in the pull-down assay. No E1 RNA was co-eluted with the Puf6-His:Loc1:She2 complex (Supplementary Figure S4), suggesting that E1 RNA does not interact with this ternary protein complex, in accordance with ChIP data on Puf6-TAP (Figure 1B). Altogether, these results show that a She2-Loc1-Puf6 ternary complex can assemble on the localization element E3, via interactions between this RNA with both She2 and Puf6.

### The interaction between Loc1 and She2 is essential for proper localization of *ASH1* mRNA

**Figure 4.**
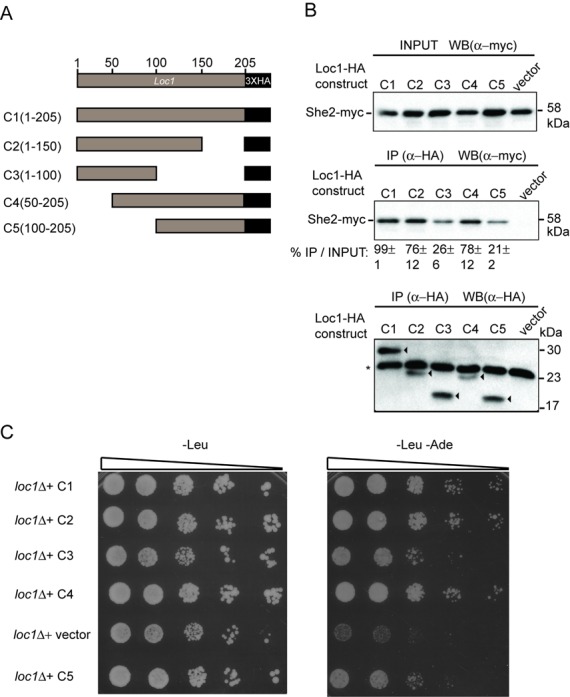
Interaction between She2 and Loc1 is important for efficient localization of *ASH1* mRNA. (**A**). Diagram of Loc1-HA constructs (C1 to C5) cloned into the YCplac111 centromeric plasmid. (**B**) Co-immunoprecipitation assay to define the interacting domain of Loc1 with She2. Using monoclonal HA.11 antibody, Loc1-HA (C1 to C5) were immunoprecipitated and the presence of She2-myc was examined by western blotting using rabbit polyclonal anti-myc antibody. Upper panel shows input for each immunoprecipitation reaction. Middle panel shows western blot of co-immunoprecipitated She2-myc along with Loc1-HA (C1 to C5). Numbers at the bottom are quantification of the percentage of IP over Input. Values presented are mean ± SEM (*N* = 3). Bottom panel correspond to the same membrane stripped and hybridized with monoclonal HA.11 antibody to show the efficiency of Loc1-HA immunoprecipitation. Arrowheads indicate different Loc1-HA constructs. Asterisk indicates light chain of IgG. (**C**) Yeast genetic assay to assess *ASH1* mRNA localization in Loc1 deletion mutants. K4452 *loc1* strain was transformed by empty vector YCPlac111 or different Loc1-HA constructs (C1 to C5). Dilutions of exponentially growing yeasts were spotted on (–Leu) and (–Leu – Ade) plates and incubated at 30°C.

Thus far, our results revealed interactions between various proteins involved in *ASH1* mRNA localization and translational control. To explore whether these interactions are important for proper localization of *ASH1* mRNA, the interaction between Loc1 (the bridging protein in the complex) and She2 was disrupted using various deletion mutants of Loc1. These deletions were fused to three HA tags and cloned in a centromeric plasmid under the control of the *LOC1* promoter (Figure 4A). The resulting plasmids were transformed into a *loc1* strain in which She2 is myc tagged. Proper expression of each Loc1 deletion construct was confirmed by western blot (Supplementary Figure S5). Using these strains, co-immunoprecipitation assays were performed to detect the interaction between the various Loc1-HA constructs and She2-myc *in vivo*. Loc1-HA constructs (C1 to C5; Figure 4A) were immunoprecipitated using anti-HA monoclonal antibody and the presence of She2-myc in the immunocomplex was measured by western blot. As shown in Figure 4B, She2-myc was immunoprecipitated along with all Loc1-HA constructs. However, after quantification of the amount of She2-myc protein present in the immune-pellets, the constructs Loc1 C3 and C5 showed dramatic reduction of the co-precipitated She2-myc protein compared to WT Loc1 (Figure 4B). These results reveal that the deletion of either half of Loc1 reduces its interaction with She2.

The Loc1-HA constructs were further tested for their effect on *ASH1* mRNA localized translation using a yeast genetic assay originally developed by Jansen and colleagues to identify the *SHE* genes (21). In this assay, the asymmetric distribution of the Ash1 protein and *HO* promoter activity was assessed using the yeast strain K4452 *loc1*, in which the *ADE2* gene is under the control of the *HO* promoter and which contains a deletion of the *LOC1* gene. In this strain, symmetric distribution of Ash1 between mother and daughter cells (due to defective *ASH1* mRNA localization and/or translation) leads to repression of the *ADE2* gene and poor growth on plate lacking adenine (-Ade). If Loc1 function is restored, Ash1 accumulates in the daughter cell, so expression of the *ADE2* gene in the mother cell allows growth on -Ade plates (Figure 4C). When transformed with a plasmid expressing the WT Loc1-HA (construct C1; Figure 4A), the K4452 *loc1* strain grew on -Leu -Ade plates, while the same strain transformed with the empty vector grew poorly (Figure 4C). Interestingly, a slower growth on -Leu -Ade of K4452 *loc1* expressing the C3 and C5 Loc1-HA constructs was observed compared to the other deletions, suggesting that these deletions disrupt Ash1 sorting. Altogether, these results suggest that the interaction between Loc1 and She2 is important for proper *ASH1* mRNA localized translation and Ash1 asymmetric distribution.

### Genome-wide identification of Puf6 interacting transcripts

**Figure 5.**
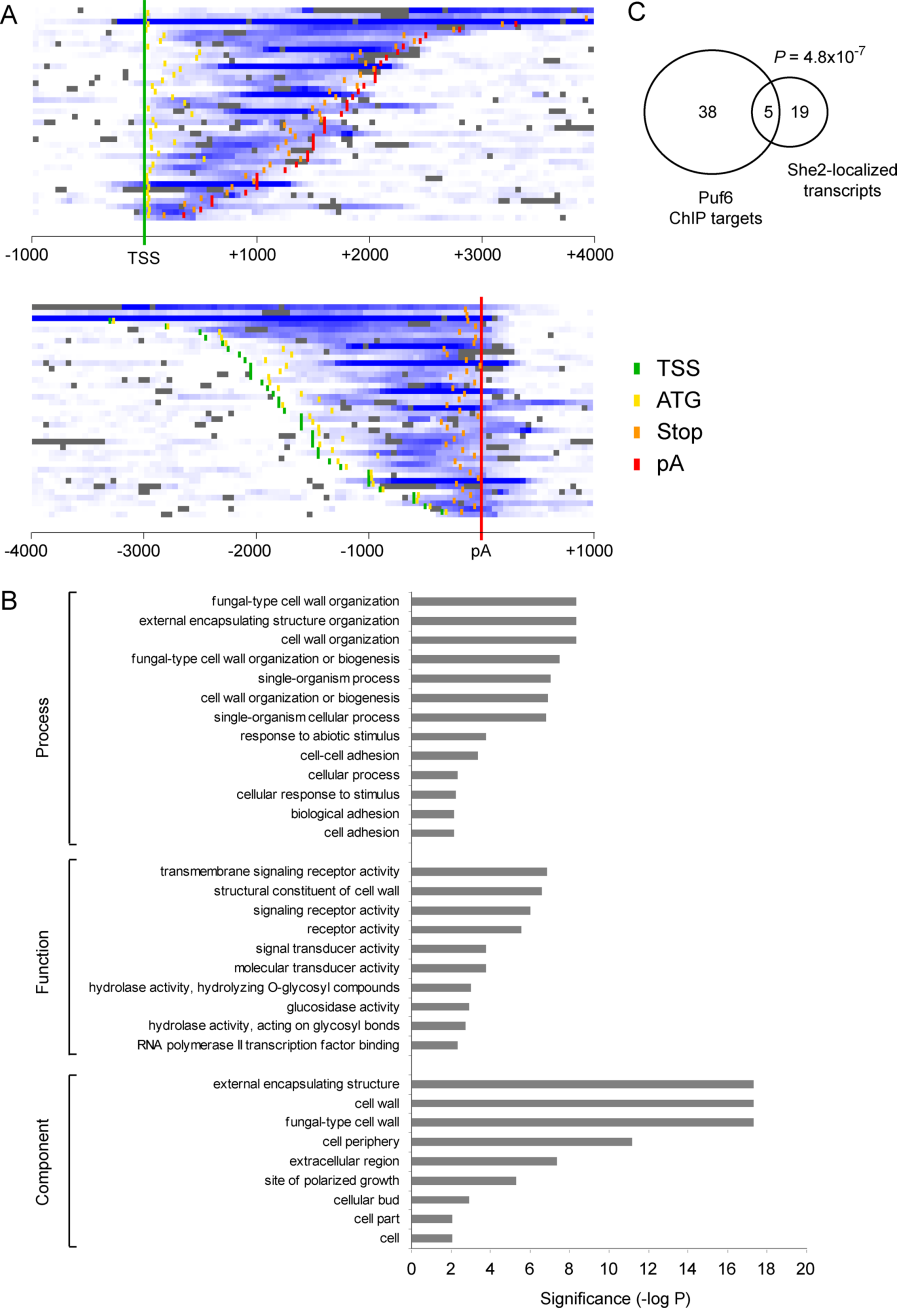
Genome-wide identification of Puf6 targets. (**A**) Heat map of Puf6 occupancy (shades of blue) along its target genes. Genes were either aligned on their TSSs (top panel) or on their pA (bottom panel). The positions for the TSS (green), ATG (yellow), stop codon (orange) and pA (red) are shown. (**B**) Gene ontologycategories that are significantly enriched among the 43 Puf6 targetgenes identified in this study. Categories with an enrichment (*P*-value) of 0.01 or less are shown. (**C**) A Venn diagram showing the overlap between the 43 Puf6 target genes identified in this study and the 24 She2-localized transcripts identified by (3).

Results presented above show that She2 physically interacts with Puf6 via Loc1 to recruit these two factors on the *ASH1* 3′UTR during transcription. Are other yeast transcripts regulated by this pathway or is this mechanism unique to *ASH1*? Previous studies have shown that Puf family proteins (Puf1–5) interact with approximately 12% of the protein coding mRNA in yeast (22). However, beside the *ASH1* mRNA, no other transcript is known to be regulated by Puf6 (8). To identify genes associated with Puf6, a genome-wide approach was used, based on ChIP-chip assay. Using a strain expressing endogenous TAP-tagged Puf6 protein, Puf6 ChIP was performed, chromatin was amplified and analyzed by tilling arrays which cover the entire yeast genome. Using this method, a total of 121 genomic regions were identified as occupied by Puf6. From these sites, 43 ORFs could be unambiguously assigned as being associated with Puf6 and were used in our analyses (Supplementary Table S4). Figure 5A represents the specific enrichment of Puf6 on its targets in the form of a heat map. Genes were either aligned on their transcription start site (TSS; top panel) or on their polyadenylation signal site (pA; bottom panel). Puf6 enrichment in most of the identified ORFs reaches its maximum toward the end of the gene but before the stop codon (orange marks), suggesting that Puf6 tends to bind before the 3′UTR.

**Figure 6.**
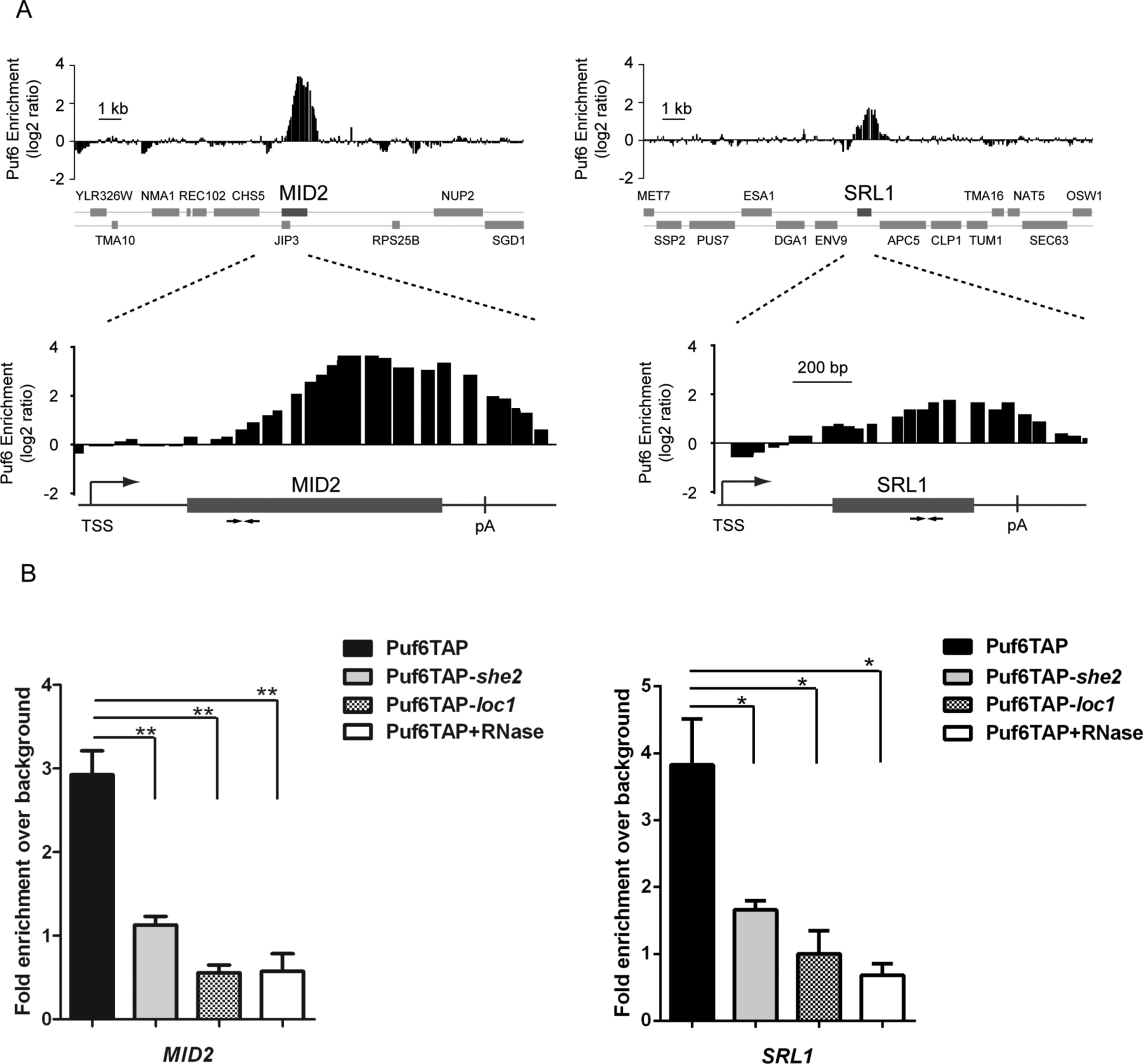
She2- and Loc1-dependent co-transcriptional recruitment of Puf6 on bud-localized mRNAs. (**A**) Genome browser snapshots of Puf6 enrichment over the regions surrounding the *MID2* and *SRL1* genes. The bottom part shows zoom-in views and arrows represent amplicons used for qPCR analysis. Amplicon lengths for MID2 and SRL1 are 92 bp and 90 bp, respectively. (**B**) ChIP-qPCR confirmation of Puf6-TAP recruitment on *MID2* and *SRL1*, in WT, *she2* and *loc1* strains. Values presented are mean ± SEM (*N* = 3) (*P < 0.05, **P < 0.01, unpaired *t*-test).

Gene ontology analysis for processes, functions and components reveals that most Puf6-associated targets encode for proteins localized to the cell periphery and are involved in cell wall biosynthesis and organization (Figure 5B). The list of newly identified genes also includes 5 out of 24 mRNAs which were shown to interact with She2 and localize to the yeast bud (Figure 5C) (3), suggesting that Puf6 plays an important role in the translational regulation of other bud-localized mRNAs (*P* = 4.8 × 10^−7^). The interaction of Puf6 with two bud-localized transcripts, *MID2* and *SRL1*, was validated by ChIP-qPCR. Figure 6A represents genomic snapshots of Puf6 enrichment at these two genes, based on our ChIP-chip data. ChIP of Puf6-TAP was performed and enrichment of specific amplicons on *MID2* and *SRL1* genes was quantified by qPCR. As shown in Figure 6B, ChIP of Puf6 generated specific signal for both genes, confirming interaction of Puf6 with these genes *in vivo.* After treatment with RNase, the ChIP signal was abolished (Figure 6B), suggesting that Puf6 interacts with these genes in a co-transcriptional manner. Finally, to explore the dependency of Puf6 recruitment on She2 and Loc1, ChIP was performed in Puf6-TAP *she2* and Puf6-TAP *loc1* strains. A significant decrease in Puf6 enrichment was observed on both genes compared to the WT strain (Figure 6B), confirming that the recruitment of Puf6 on *MID2* and *SRL1* is She2- and Loc1-dependent.

## DISCUSSION

The current paradigm in the field of mRNA localization posits that mRNA localization is coupled to the translational repression of transcripts during their transport (23). While some RNA-binding proteins, such as ZBP1, combine both localization and translational repression activities (24), in most cases, the two are performed by independent *trans-*acting factors (25). Therefore, this raises the question of whether the recruitment of the localization and translational repression machineries on a given mRNA occurs independently, or if their assembly depends on each other. Evidence for co-dependency between these factors comes from yeast, in which previous studies have shown that the nuclear targeting of the mRNA localization factor She2 is important for the binding of the translational repressor Puf6 on *ASH1* mRNA (13). In the absence of She2 in the nucleus, *ASH1* mRNA localization defects and premature *ASH1* mRNA translation have been observed (12,13).

**Figure 7.**
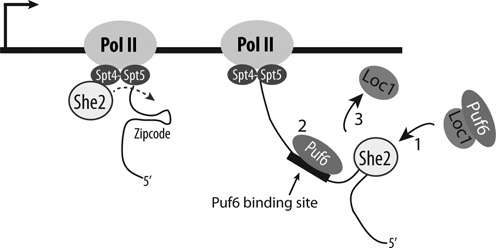
Model for the recruitment of Puf6 on bud-localized mRNAs. After being co-transcriptionally loaded on the elongating mRNA via She2 and Loc1 (1), Puf6 is transferred to the nascent mRNA via an interaction with its binding site (2). Since Loc1 never leaves the nucleus (10), it should be separated from the complex (3) before the mRNP complex is exported to the cytoplasm.

In this study, we describe a novel mechanism allowing the functional coupling between mRNA localization and translational control factors on localized mRNAs. We show that the translational repressor Puf6 is recruited co-transcriptionally on its target mRNAs via an interaction with the mRNA localization factor She2, mediated by the protein Loc1 (Figure 7). We provide evidence of a ternary complex formed between She2, Loc1 and Puf6, which explains how recruitment of Puf6 on the 3′UTR of *ASH1* mRNA is dependent on Loc1 and She2. The dependency of Puf6 recruitment on Loc1 emphasizes a direct role for Loc1 in coupling mRNA localization and translational control. Current genetic data supports this model, since the deletion of *LOC1* phenocopy a *puf6* KO, as both mutants disrupt *ASH1* mRNA localization (8,10), and increase *ASH1* mRNA translation (11,12). It was previously suggested that Loc1 acts on *ASH1* mRNA translation by mediating the synthesis of ‘specialized ribosomes’ (11). Although we cannot exclude this possibility, our results point toward a primary function for this protein as a loading factor for Puf6. This novel function of Loc1 is crucial for a complete assembly of RNA localization factors on *ASH1* mRNA, as we showed that Puf6 interaction with its RNA motif is limited *in vivo* in the absence of both She2 and Loc1.

Our results are in agreement with a recent study published while this manuscript was in revision, which also report a direct interaction between She2 and Loc1 *in vitro* (26). However, no direct interaction between recombinant Puf6 and Loc1 was detected in this study, and the authors did not observe the formation of a Puf6:Loc1:She2 ternary complex on the E3 RNA zipcode *in vitro*. While these results await *in vivo* validation, experimental conditions and differences in protein tagging may explain the divergences between our studies. Our finding of a co-transcriptional recruitment of Puf6 by Loc1 also challenges a previous model suggesting that Puf6 and Loc1 are loaded post-transcriptionally on *ASH1* mRNA in the nucleolus prior to its export to the cytoplasm (12). Evidence for this nucleolar transition comes from an experiment showing that upon blocking nuclear export, *ASH1* mRNA accumulates in the nucleolus in a She2-dependent manner (12). However, our study does not eliminate the possibility that *ASH1* must pass through the nucleolus before being exported to the cytoplasm.

Using ChIP-chip, we show that Puf6 is loaded co-transcriptionally on other target mRNAs and, at least in the case of bud-localized mRNAs, this loading is dependent on both She2 and Loc1. Unlike ChIP-qPCR results, binding pattern of ChIP-chip data shows an enrichment signal which spreads over a region on identified genes, a profile which may suggest an interaction of Puf6 with the transcriptional machinery. However, immunoprecipitation of Spt4 and Spt5 results in an RNase sensitive co-purification of Puf6 (Supplementary Figure S6), excluding the possibility of a direct interaction between Puf6 and elongating RNA polymerase. Thus, we assume that the observed discrepancy in enrichment pattern of Puf6 on *ASH1* by ChIP-qPCR and newly identified targets by ChIP-chip is likely due to differences in the ChIP protocols used.

Analysis of the ChIP-chip results reveals that Puf6 binds preferentially to mRNA encoding factors involved in cell wall biogenesis. For instance, Puf6 binds to the transcripts coding for four of the five cell wall stress sensors: *WSC2* and *3, MTL1* and *MID2* (27), suggesting that Puf6 may coordinate the regulation of these proteins. Interestingly, Puf6 targets largely overlap with the known mRNA targets of Khd1, including a preferential association with mRNAs encoding for cell wall proteins (28,29). These results reveal a very close functional relationship between these two translational repressors, as previously suggested by their common role in regulating *ASH1* mRNA translation.

Finally, the coordinated mechanism of Puf6 recruitment is reminiscent of ZBP1 loading on β-actin mRNA, which localizes this transcript at the leading edge of fibroblasts (30). ZBP1 is recruited co-transcriptionally on nascent β-actin mRNA and its loading requires prior recruitment of the predominately nuclear protein ZBP2 on a site near ZBP1-binding element or zipcode (31,32). Like ZBP2, Loc1 dissociates from the localization complex and is not exported to cytoplasm. While no direct interaction has been reported between ZBP1 and ZBP2, this reveals that such coordinated assembly is also used on localized mRNA in metazoans. In a more general perspective, recent evidence suggests that the cytoplasmic fate of an mRNA (its cytoplasmic localization and decay) can be primed on nascent transcripts (5,33). Our results further broaden this perspective by showing that translational regulation is also established on nascent mRNAs, and that mRNA transcription is a process during which mechanisms of post-transcriptional gene regulation can be coordinated.

## SUPPLEMENTARY DATA

Supplementary Data are available at NAR Online, including [34,35]

SUPPLEMENTARY DATA

## References

[1] Martin K.C., Ephrussi A. (2009). mRNA localization: gene expression in the spatial dimension. Cell.

[2] Shahbabian K., Chartrand P. (2012). Control of cytoplasmic mRNA localization. Cell Mol. Life Sci..

[3] Shepard K.A., Gerber A.P., Jambhekar A., Takizawa P.A., Brown P.O., Herschlag D., DeRisi J.L., Vale R.D. (2003). Widespread cytoplasmic mRNA transport in yeast: identification of 22 bud-localized transcripts using DNA microarray analysis. Proc. Natl Acad. Sci. U.S.A..

[4] Long R.M., Singer R.H., Meng X., Gonzalez I., Nasmyth K., Jansen R.P. (1997). Mating type switching in yeast controlled by asymmetric localization of ASH1 mRNA. Science.

[5] Shen Z., St-Denis A., Chartrand P. (2010). Cotranscriptional recruitment of She2p by RNA pol II elongation factor Spt4-Spt5/DSIF promotes mRNA localization to the yeast bud. Genes Dev..

[6] Muller M., Heym R.G., Mayer A., Kramer K., Schmid M., Cramer P., Urlaub H., Jansen R.P., Niessing D. (2011). A cytoplasmic complex mediates specific mRNA recognition and localization in yeast. PLoS Biol..

[7] Takizawa P.A., Vale R.D. (2000). The myosin motor, Myo4p, binds Ash1 mRNA via the adapter protein, She3p. Proc. Natl Acad. Sci. U.S.A..

[8] Gu W., Deng Y., Zenklusen D., Singer R.H. (2004). A new yeast PUF family protein, Puf6p, represses ASH1 mRNA translation and is required for its localization. Genes Dev..

[9] Deng Y., Singer R.H., Gu W. (2008). Translation of ASH1 mRNA is repressed by Puf6p-Fun12p/eIF5B interaction and released by CK2 phosphorylation. Genes Dev..

[10] Long R.M., Gu W., Meng X., Gonsalvez G., Singer R.H., Chartrand P. (2001). An exclusively nuclear RNA-binding protein affects asymmetric localization of ASH1 mRNA and Ash1p in yeast. J. Cell Biol..

[11] Komili S., Farny N.G., Roth F.P., Silver P.A. (2007). Functional specificity among ribosomal proteins regulates gene expression. Cell.

[12] Du T.G., Jellbauer S., Muller M., Schmid M., Niessing D., Jansen R.P. (2008). Nuclear transit of the RNA-binding protein She2 is required for translational control of localized ASH1 mRNA. EMBO Rep..

[13] Shen Z., Paquin N., Forget A., Chartrand P. (2009). Nuclear shuttling of She2p couples ASH1 mRNA localization to its translational repression by recruiting Loc1p and Puf6p. Mol. Biol. Cell.

[14] Lindstrom D.L., Squazzo S.L., Muster N., Burckin T.A., Wachter K.C., Emigh C.A., McCleery J.A., Yates J.R., Hartzog G.A. (2003). Dual roles for Spt5 in pre-mRNA processing and transcription elongation revealed by identification of Spt5-associated proteins. Mol. Cell Biol..

[15] Longtine M.S., McKenzie A., Demarini D.J., Shah N.G., Wach A., Brachat A., Philippsen P., Pringle J.R. (1998). Additional modules for versatile and economical PCR-based gene deletion and modification in Saccharomyces cerevisiae. Yeast.

[16] Bataille A.R., Jeronimo C., Jacques P.E., Laramee L., Fortin M.E., Forest A., Bergeron M., Hanes S.D., Robert F. (2012). A universal RNA polymerase II CTD cycle is orchestrated by complex interplays between kinase, phosphatase, and isomerase enzymes along genes. Mol. Cell.

[17] Boyer L.A., Lee T.I., Cole M.F., Johnstone S.E., Levine S.S., Zucker J.P., Guenther M.G., Kumar R.M., Murray H.L., Jenner R.G. (2005). Core transcriptional regulatory circuitry in human embryonic stem cells. Cell.

[18] Olivier C., Poirier G., Gendron P., Boisgontier A., Major F., Chartrand P. (2005). Identification of a conserved RNA motif essential for She2p recognition and mRNA localization to the yeast bud. Mol. Cell Biol..

[19] Niessing D., Huttelmaier S., Zenklusen D., Singer R.H., Burley S.K. (2004). She2p is a novel RNA binding protein with a basic helical hairpin motif. Cell.

[20] Ghaemmaghami S., Huh W.K., Bower K., Howson R.W., Belle A., Dephoure N., O'Shea E.K., Weissman J.S. (2003). Global analysis of protein expression in yeast. Nature.

[21] Jansen R.P., Dowzer C., Michaelis C., Galova M., Nasmyth K. (1996). Mother cell-specific HO expression in budding yeast depends on the unconventional myosin myo4p and other cytoplasmic proteins. Cell.

[22] Gerber A.P., Herschlag D., Brown P.O. (2004). Extensive association of functionally and cytotopically related mRNAs with Puf family RNA-binding proteins in yeast. PLoS Biol..

[23] Besse F., Ephrussi A. (2008). Translational control of localized mRNAs: restricting protein synthesis in space and time. Nat. Rev. Mol. Cell Biol..

[24] Huttelmaier S., Zenklusen D., Lederer M., Dictenberg J., Lorenz M., Meng X., Bassell G.J., Condeelis J., Singer R.H. (2005). Spatial regulation of beta-actin translation by Src-dependent phosphorylation of ZBP1. Nature.

[25] Vazquez-Pianzola P., Suter B. (2012). Conservation of the RNA transport machineries and their coupling to translation control across eukaryotes. Comp. Funct. Genomics.

[26] Niedner A., Müller M., Moorthy B.T., Jansen R.-P., Niessing D. (2013). Role of Loc1p in assembly and reorganization of nuclear ASH1 messenger ribonucleoprotein particles in yeast. Proc. Natl Acad. Sci. U.S.A..

[27] Levin D.E. (2011). Regulation of cell wall biogenesis in Saccharomyces cerevisiae: the cell wall integrity signaling pathway. Genetics.

[28] Hasegawa Y., Irie K., Gerber A.P. (2008). Distinct roles for Khd1p in the localization and expression of bud-localized mRNAs in yeast. RNA.

[29] Wolf J.J., Dowell R.D., Mahony S., Rabani M., Gifford D.K., Fink G.R. (2010). Feed-forward regulation of a cell fate determinant by an RNA-binding protein generates asymmetry in yeast. Genetics.

[30] Ross A.F., Oleynikov Y., Kislauskis E.H., Taneja K.L., Singer R.H. (1997). Characterization of a beta-actin mRNA zipcode-binding protein. Mol. Cell Biol..

[31] Oleynikov Y., Singer R.H. (2003). Real-time visualization of ZBP1 association with beta-actin mRNA during transcription and localization. Curr. Biol..

[32] Pan F., Huttelmaier S., Singer R.H., Gu W. (2007). ZBP2 facilitates binding of ZBP1 to beta-actin mRNA during transcription. Mol. Cell Biol..

[33] Haimovich G., Choder M., Singer R.H., Trcek T. (2013). The fate of the messenger is pre-determined: a new model for regulation of gene expression. Biochim. Biophys. Acta.

[34] Gietz R.D., Sugino A. (1988). New yeast-Escherichia coli shuttle vectors constructed with in vitro mutagenized yeast genes lacking six-base pair restriction sites. Gene.

[35] Long R.M., Gu W., Lorimer E., Singer R.H., Chartrand P. (2000). She2p is a novel RNA-binding protein that recruits the Myo4p-She3p complex to ASH1 mRNA. EMBO J..

