# Supplementary material for: Co-transcriptional recruitment of Puf6 by She2 couples translational repression to mRNA localization
Source: Nucleic Acids Res. 2014 Jul 9;42(13):8692–704. doi: 10.1093/nar/gku597 (PMC4117797; doi:10.1093/nar/gku597)
Supplement: SUPPLEMENTARY DATA [file supp_42_13_8692__index.html]

Co-transcriptional recruitment of Puf6 by She2 couples translational repression to mRNA localization — Co-transcriptional recruitment of Puf6 by She2 couples translational repression to mRNA localization — SUPPLEMENTARY DATA 

# Co-transcriptional recruitment of Puf6 by She2 couples translational repression to mRNA localization

## SUPPLEMENTARY DATA

**Files in this Data Supplement:**

- Supplementary Data 1
- Supplementary Data 2
