# Supplementary material for: Co-transcriptional recruitment of Puf6 by She2 couples translational repression to mRNA localization
Source: Nucleic Acids Res. 2014 Jul 9;42(13):8692–704. doi: 10.1093/nar/gku597 (PMC4117797; doi:10.1093/nar/gku597)
Supplement: SUPPLEMENTARY DATA [file supp_gku597_nar-03537-a-2013-File009.pdf]

Supplementary information for:

Co-transcriptional recruitment of Puf6 by She2 couples translational repression to mRNA localization

Karen Shahbabian, Célia Jeronimo, Amélie Forget, François Robert, Pascal Chartrand

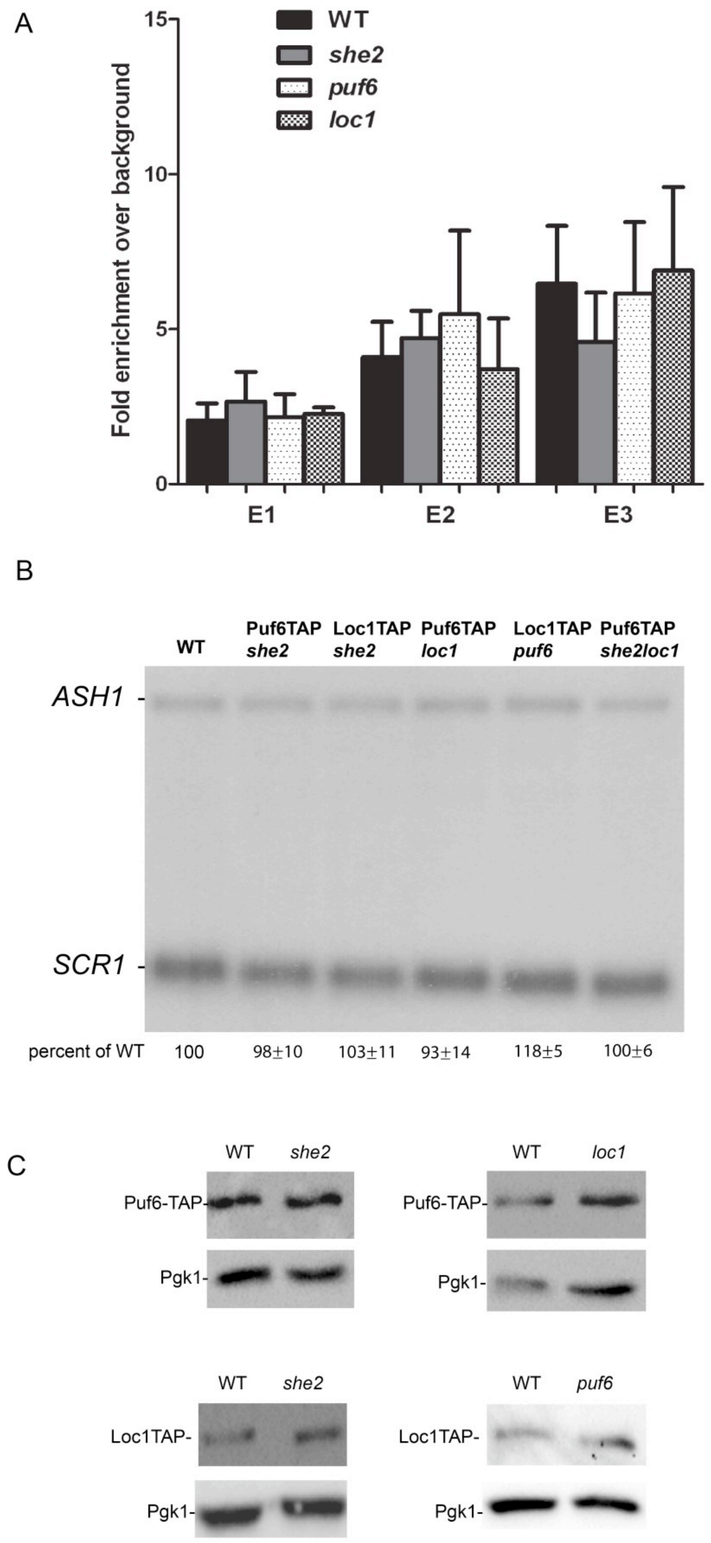
A
WT
she2
puf6
loc1
Fold enrichment over background
15
10
5
0
E1
E2
E3
B
WT
Puf6TAP she2
Loc1TAP she2
Puf6TAP loc1
Loc1TAP puf6
Puf6TAP she2loc1
ASH1
SCR1
percent of WT
100
98±10
103±11
93±14
118±5
100±6
C
WT
she2
loc1
puf6
Puf6-TAP
Loc1TAP
Pgk1

Supplementary Figure S1: Effect of *she2*, *loc1* or *puf6* mutants on *ASH1* transcription, *ASH1* mRNA levels and TAP-tagged protein expression levels. **A)** ChIP performed with monoclonal 8WG16 antibody. After ChIP, enrichment of E1, E2B and E3 amplicons were quantified by q-PCR. *SCR1* which is transcribed by RNA Pol III was used as background. Data presented are mean ± SEM (N=3). **B)** Northern blot performed on RNAs extracted from wild-type and mutant strains. *ASH1* mRNA level was quantified and normalized to *SCR1* mRNA. Numbers at the bottom of the gel is percentage of *ASH1* mRNA comparing to wild-type strain**. C)** Western blot analysis of Puf6-TAP and Loc1-TAP in wild-type and mutant strains. Upper panels are western blot detection of Puf6-TAP in WT, *she2* and *loc1* strains. Lower panels are western blot of Loc1-TAP in WT, *she2* and *puf6* strains.

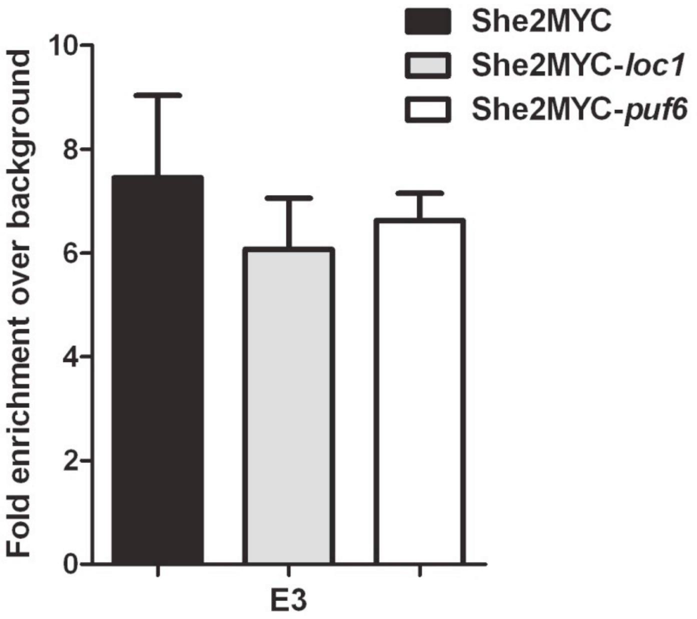

Supplementary Figure S2: The recruitment of She2 is independent of Loc1 and Puf6: ChIP of She2-myc was performed using monoclonal 9E10 antibody, and enrichment of She2-myc on E3 element, in wild type and mutant strains was quantified using q-PCR. Data presented are mean ± SEM (N=3).

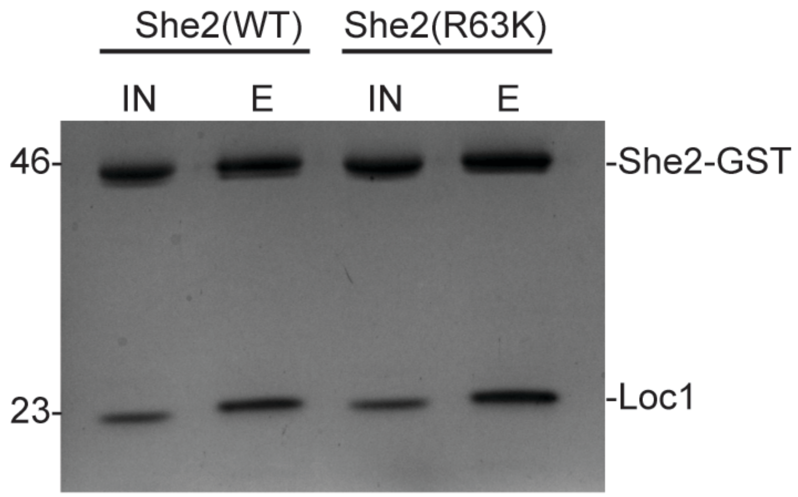

Supplementary Figure S3: She2WT and She2R63K mutant interact with same efficiency with Loc1. GST pull down experiment performed by purified She2-GST (WT and R63K mutant) and Loc1 recombinant protein. Note that same amount of Loc1 protein is eluted from WT or mutant She2-GST.

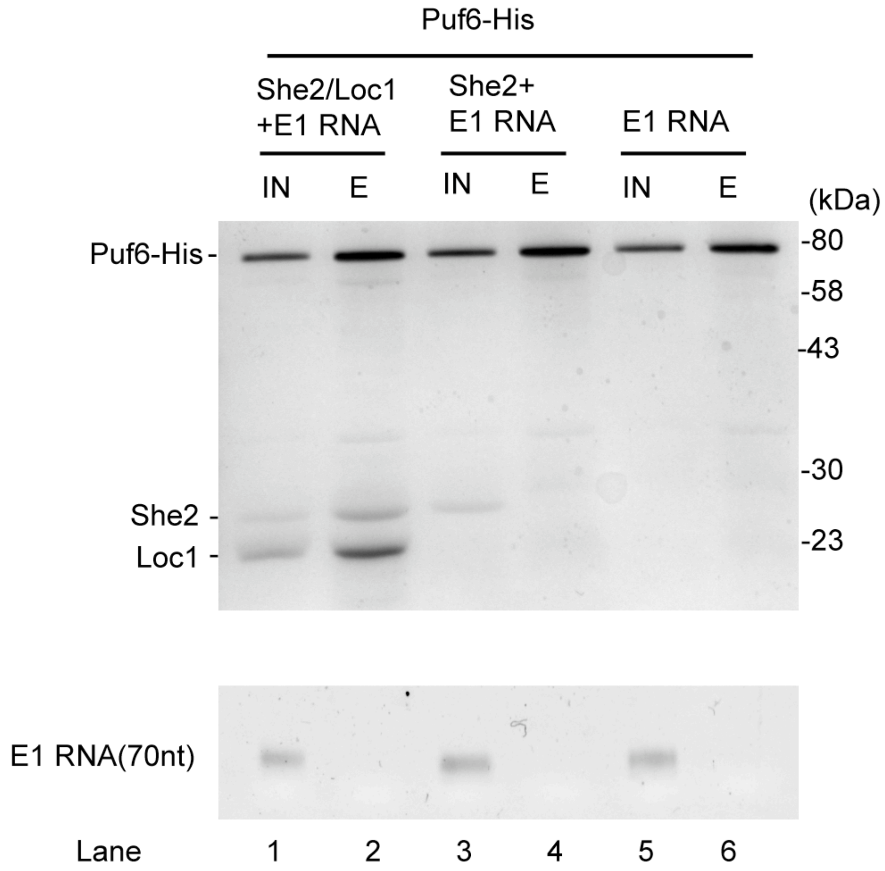

Supplementary Figure S4: *in vitro* reconstitution assay of E1-mRNP. Pull down experiment with Puf6-His, Loc1, She2 and 70 nt E1 RNA. Note that RNA was not eluted from neither of complexes showing that there is no interaction between E1 RNA and Puf6-His or She2. Upper gel corresponds to protein gel stained by Coomassie blue, lower gel is RNA PAGE stained by Gel-RED (IN=Input, E=Elution).

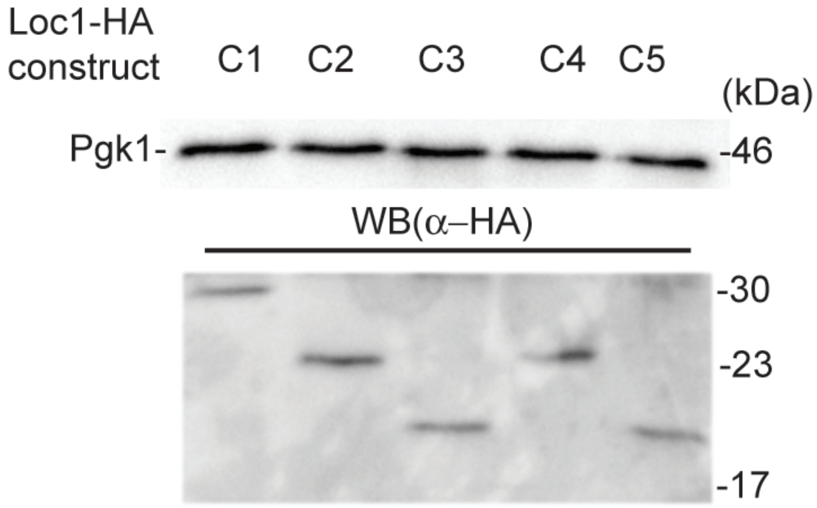

Supplementary Figure S5: Expression level of Loc1-HA constructs (C1 to C5) in BY4741 *loc1* She2-myc strain. Upper panel is western blot detection of Pgk1 using anti-Pgk1 antibody. Lower panel correspond to western blot of Loc1-HA using anti-HA antibody.

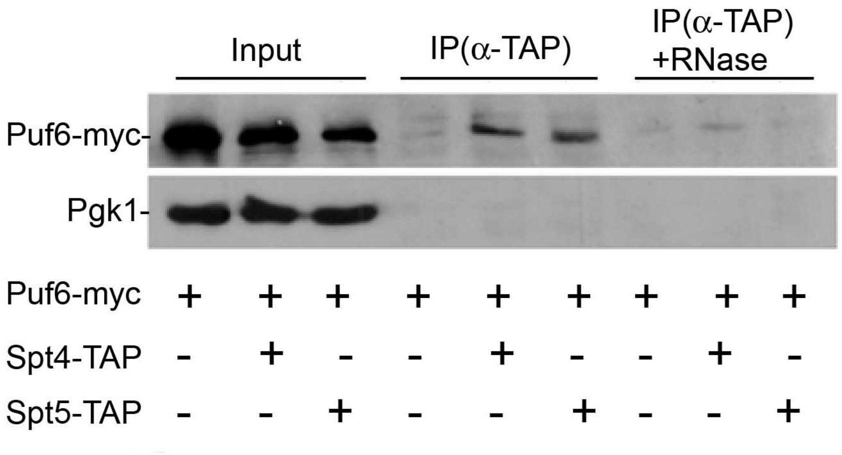

Supplementary Figure S6: Puf6 interacts with Spt4-Spt5 via RNA. Co-immunoprecipitation assay to explore the interaction of Puf6-myc with Spt4-TAP and Spt5-TAP. Note that after RNase treatment the interaction is abolished.

Supplementary Table S1. Strains used in this study

Strain	Genotype	Source
BY4741	*Mat a, his3Δ1, leu2Δ0, met15Δ0, ura3Δ0*	Open Biosystems
BY4741 *she2*	BY4741 *she2::KAN*	Open Biosystems
Puf6-TAP	BY4741 *PUF6-TAP::HIS3*	Open Biosystems
Loc1-TAP	BY4741 *LOC1-TAP::HIS3*	Open Biosystems
Puf6-TAP *she2*	BY4741 *PUF6-TAP::HIS3 she2::KAN*	This study
Puf6-TAP *loc1*	BY4741 *PUF6-TAP::HIS3 loc1::URA3*	This study
Puf6-TAP *she2 loc1*	BY4741 *PUF6-TAP::HIS3 she2::KAN loc1::URA3*	This study
Loc1-TAP *she2*	BY4741 *LOC1-TAP::HIS3 she2::KAN*	This study
Loc1-TAP *puf6*	BY4741 *LOC1-TAP::HIS3 puf6::KAN*	This study
Puf6-TAP She2MYC	BY4741 *PUF6-TAP::HIS3 she2::KAN* + YCP111She2MYC	This study
Puf6-TAP She2R63KMYC	BY4741 *PUF6-TAP::HIS3 she2::KAN* + YCP111She2R63KMYC	This study
Puf6-TAP *loc1* She2MYC	BY4741 *PUF6-TAP::HIS3 she2::KAN loc1::URA3* + YCP111She2MYC	This study
Puf6-TAP Loc1-HA	BY4741 *PUF6-TAP::HIS3 LOC1-HA::Leu2*	This study
Puf6-TAP *she2* Loc1-HA	BY4741 *PUF6-TAP::HIS3 she2::KAN LOC1-HA::Leu2*	This study
Loc1-TAP She2MYC	BY4741 *LOC1-TAP::HIS3* + YCP111She2MYC	This study
Loc1-TAP She2R63KMYC	BY4741 *LOC1-TAP::HIS3* + YCP111-She2R63K-MYC	This study
She2-MYC	BY4741 *SHE2-13xMYC::HIS3*	This study
She2-MYC *loc1*	BY4741 *SHE2-13xMYC::HIS3 loc1::KAN*	This study
She2-MYC *puf6*	BY4741 *SHE2-13xMYC::HIS3 puf6::KAN*	This study
BY4741 *ash1*	BY4741 *ash1::KAN*	This study
BY4741 *ASH1* WT	BY4741 *ash1::KAN* + YIP128-*ASH1* WT	This study
BY4741 *ASH1* 3'UTR mutated	BY4741 *ash1::KAN* + YIP128-*ASH1* 3'UTR mutated	This study
BY4741 *loc1* She2-MYC	BY4741 *loc1::KAN SHE2-13xMYC::HIS3*	This study
She2-MYC Loc1-HA (C1)	BY4741 *loc1* She2MYC + YCP111-*LOC1*-C1	This study
She2-MYC Loc1-HA (C2)	BY4741 *loc1* She2MYC + YCP111-*LOC1*-C2	This study
She2-MYC Loc1-HA (C3)	BY4741 *loc1* She2MYC + YCP111-*LOC1*-C3	This study
She2-MYC Loc1-HA (C4)	BY4741 *loc1* She2MYC + YCP111-*LOC1*-C4	This study
She2-MYC Loc1-HA (C5)	BY4741 *loc1* She2MYC + YCP111-*LOC1*-C5	This study
K4452 *loc1*	*Mat α, his3, leu2, ade2, trp1, ura3, HO-ADE2, HO-CAN1, loc1::NAT*	This study
*loc1*Δ+ C1	K4452 *loc1* +YCP111-*LOC1*-C1	This study

*loc1*Δ+ C2	K4452 *loc1* + YCP111-*LOC1*-C2	This study
*loc1*Δ+ C3	K4452 *loc1* + YCP111-*LOC1*-C3	This study
*loc1*Δ+ C4	K4452 *loc1* + YCP111-*LOC1*-C4	This study
*loc1*Δ+ C5	K4452 *loc1* + YCP111-*LOC1*-C5	This study

Supplementary Table S2. Plasmids used in this study

pGEX-6P1	Vector for the expression of GST fused proteins with PreScission cleavage site	GE healthcare
pGEX-4T3	Vector for the expression of GST fused proteins with Thrombin cleavage site	GE healthcare
YCPlac111	Single copy yeast vector with *LEU2* selectable marker	(34)
YIP128	Yeast integrative vector with *LEU2* selectable marker	(34)
pGEX-6P1-SHE2	Vector for expression of She2-GST	(5)
pGEX-6P1-SHE2(R63K)	Vector for expression of She2(R63K)-GST	This study
pGEX-6P1-LOC1	Vector for expression of Loc1-GST	This study
pGEX-4T3-PUF6-His	Vector for expression of Puf6 with N-terminal GST tag and C-terminal His-tag	This study
pGEM4Z1-E1	For *in vitro* transcription of E1 element	(18)
pRL168	For *in vitro* transcription of E3 element	(35)
pGEM4Z1-mE3	For *in vitro* transcription of mutated E3 element	This study
YCP111-SHE2MYC	Expresses She2-9xmyc from endogenous promoter	This study
YCP111-SHE2R63KMYC	Expresses She2R63K-9xmyc from endogenous promoter	This study
YIP128-*ASH1*WT	Integrative plasmid used for the integration of WT *ASH1* in *LEU2* locus	This study
YIP128-*ASH1* 3’UTR mutated	Integrative plasmid used for the integration of 3’UTR mutated *ASH1* in *LEU2* locus	This study
YCP111-LOC1-C1	YCplac111 vector for expression of Loc1-HA (amino acids 1-205) from *LOC1* promoter	This study
YCP111-LOC1-C2	YCplac111 vector for expression of Loc1-HA (amino acids 1-150) from *LOC1* promoter	This study
YCP111-LOC1-C3	YCplac111 vector for expression of Loc1-HA (amino acids 1-100) from *LOC1* promoter	This study
YCP111-LOC1-C4	YCplac111 vector for expression of Loc1-HA (amino acids 50-205) from *LOC1* promoter	This study
YCP111-LOC1-C5	YCplac111 vector for expression of Loc1-HA (amino acids 100-205) from *LOC1* promoter	This study

Supplementary Table S3. Primers used for ChIP

Primer	Sequence
E1-for	TGGAAAACTGATCTTACCCATTG
E1-Rev	TTGGGTATACTTAATGGCTTGAAA
E2B-for	CGACAACGCTAGTTACAG
E2B-Rev	TTGGGCTTGGAGTGTATG
E3-for	TCGTAAGATCCCCACAAAGG
E3-Rev	AACGGTACCCTTCAATTTCG
Intergenic-for	TAGGGCACGTACATGGCAAC
Intergenic-rev	CATCGTCATCACGCTTCATC
SCR1-for	TCTGGTGGGATGGGATAC
SCR1-rev	CGGTGCGGAATAGAGAAC
MID2-for	GAGTTCGTCTACCAGCATTG
MID2-rev	CTGAAGAAGCAGAGGAAGAG
SRL1-for	CTGCAGCAAGTGTCAGTG
SRL1-rev	GGACGCAACCATTGGAAG
